# Risk in synthetic biology—views from the lab: Early career scientists’ concerns about synthetic biology open up new perspectives on risk and responsibility in research

**DOI:** 10.15252/embr.201845958

**Published:** 2018-06-01

**Authors:** Carmen McLeod, Stevienna de Saille, Brigitte Nerlich

**Affiliations:** ^1^ University of Nottingham Nottingham UK; ^2^ University of Sheffield Sheffield UK

**Keywords:** S&S: Careers & Training, S&S: Ethics, Synthetic Biology & Biotechnology

## Abstract

Gauging young scientists’ concerns about and views of research in synthetic biology opens up new perspectives on career paths, economic expectations and mental health issues in cutting‐edge research.

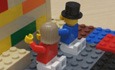

The concepts of risk and responsibility are often linked to discussions of emerging scientific fields, but studies into how these concepts are connected to research practices have been narrowly focused on risks for humans and the environment. To broaden these concepts, “Responsible Research and Innovation” (RRI), a democratic governance framework, aims to enable societal discussions beyond traditional risk assessment and mitigation. Proponents of RRI argue that these discussions should not be confined to the direct risks of the research itself, but also include wider issues, such as “the purposes and motivations of research” 1. Yet, it is not only RRI protagonists who want to broaden this conversation. We found that scientists also ponder non‐technical risks, such as the impact of institutional demands on career, health and social relationships, or economic pressures from the incentive system in which much of research in biology is now embedded. These findings challenge the present formulation of RRI as a science governance framework and lead us to argue that “responsible” research and innovation systems can only succeed if these broader concerns are taken as seriously as the risk of laboratory accident or inadvertent release.

## Risks and responsibility

Synthetic biology has been heralded as a new technology to provide innovative technological solutions for global environmental and health challenges. Risks related to the field have generally been discussed in the context of technical risks, such as accidental release of artificial organisms into the environment or the dangers of do‐it‐yourself (DIY) biology and the easy availability of materials via the Internet, which could be misused for bioterrorism. It has also included some discussion of trade and social justice issues 2. Responsible Research and Innovation is therefore seen as a strategy to link technical and societal concerns 3 and encourage scientists to anticipate, discuss, reflect and act upon risk in open, transparent and inclusive ways, from the beginning of a research project all the way to the market. Some funders and policymakers have claimed that RRI will accelerate the successful development of new technologies, while simultaneously ensuring that this will be done “responsibly” 4.

**…** scientists also ponder non‐technical risks, such as the impact of institutional demands on career, health and social relationships, or economic pressures…

In the UK, six synthetic biology research centres were recently created at the Universities of Nottingham, Cambridge, Bristol, Manchester, Warwick and Edinburgh, funded by Research Councils UK with currently more than £60 million. These centres have been tasked with embedding the principles of RRI into their research and innovation processes to open up their research activities to societal discussion at an early stage. It is hoped that this will help to identify emerging issues and concerns so as to steer or shape innovation pathways, ensuring that they are socially desirable and in the public interest 1, 5.

## Modelling risks in a new way

Against this background, we convened a series of workshops with PhD students, postdoctoral researchers and technicians from one of the UK Synthetic Biology research centres to encourage participants to reflect on risks and responsibilities in their research, but without predefining what “risk” might be. We used LEGO^®^ SERIOUS PLAY^®^ (LSP), a novel method that uses specialised sets of bricks with a trained facilitator (de Saille). The central idea of LSP is that making a physical representation of the response to a question stimulates a deeper level of creative thinking 6, while using visual and verbal metaphors to tell a responsive story through the model allows tacit knowledge and values to emerge.

The workshops began with a set of exercises to familiarise participants with the process, after which they were asked to build responses to questions about what they loved about science, what they understood to be the biggest risk in their work and what might be done to mitigate it. Using this method, we found that most of the researchers came up with responses that we could not have anticipated, and which did not match either traditional conceptions of risk assessment or emerging ideas about responsible research and innovation.

We had anticipated that most participants would identify technological or scientific risks amenable to assessment and management, and some did indeed follow this pattern. Across all six groups of 10–12 participants, we consistently found that the most frequently articulated risks were not literal and physical risks in the laboratory, such as explosions or accidental release, or the risk of public rejection outside the laboratory—although these were all mentioned at least once in most of the groups. Instead, to our surprise, the majority of participants focused either on personal risk to their own mental health or career, societal risk in relying upon a technological “fix” or more ephemeral risks to science as the pursuit of knowledge. They interpreted “responsibility” as a difficult path they had to navigate between economic expectations, work–life realities, and the particular difficulties of cutting‐edge science.

**Figure 1:**
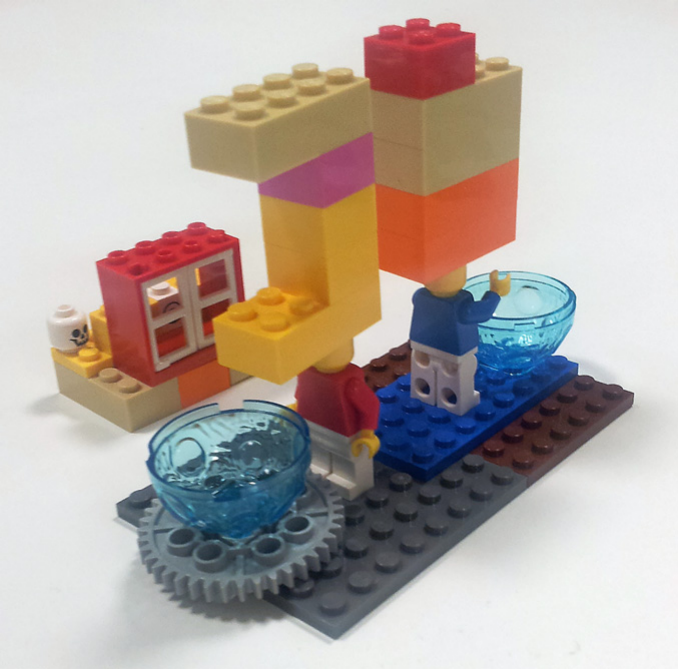
Societal expectations “You've got two scientists, they're both working on the same thing, but they're not talking to each other; their backs are turned to each other, and they both have loads of bricks on their head, because of the amount of pressure that's put on scientists to produce and publish and all the rest of it. And you've got all the people watching them, waiting for them to publish, so they're going to start making up results, and lying and things. I think that's one of the biggest problems we have in science.”

The workshop began by asking participants what they loved most about science. Overwhelmingly, they emphasised “figuring out how things work”, finding greener/more sustainable ways of doing things and the collaborative process of research. The values encoded in these responses echoed throughout the questions focussing on risk, with many respondents modelling the ways in which the increased stress and pressure of doing this form of highly interdisciplinary research could warp the research process, as seen in Fig 1.

Some funders and policymakers have claimed that RRI will accelerate the successful development of new technologies, while simultaneously ensuring that this will be done “responsibly”

Using the metaphor of a pile of bricks pressing down on researchers, this model represents the participant's worry that such pressures can force scientists to falsify results in order to get publications. This participant was also concerned that, as funding for basic science increasingly depends on industry, scientists are not only less able to collaborate, but are also less free to discuss their work and results, further complicating the burden of expectation on researchers in these fields.

**Figure 2:**
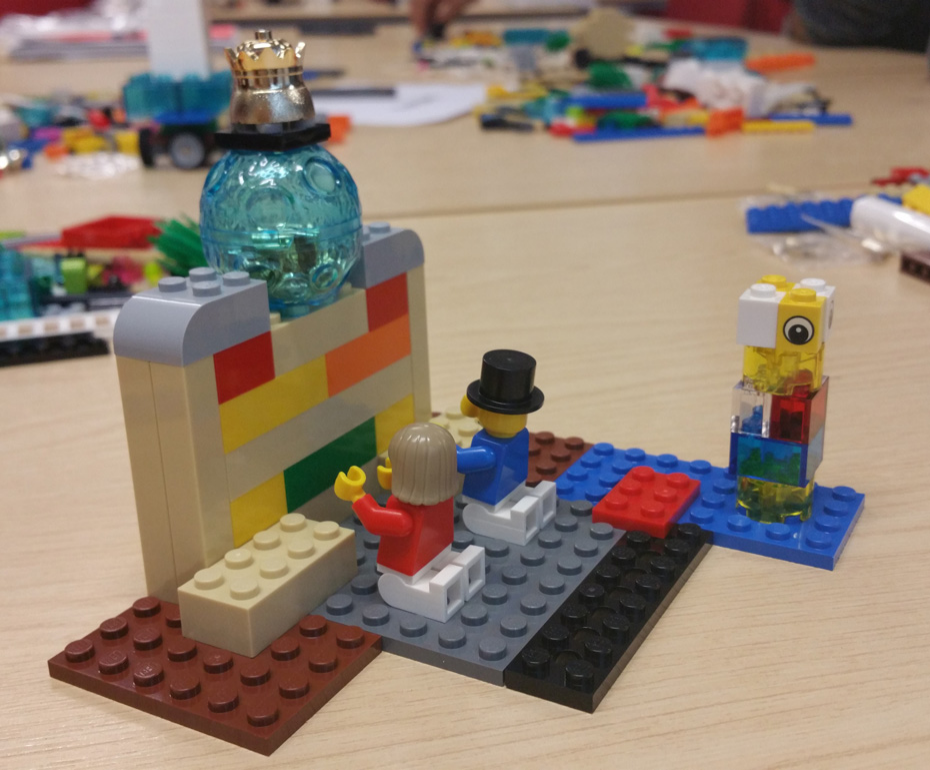
Economic incentives “[P]eople [are] always looking forward and looking up to what is the financial ideal, what is the market, or what can we patent, what can we get money for? Whereas the strange animal that is academic and socially valuable research is left in the corner”.

A number of participants addressed similar risks arising from the profit motive, worrying that this could destroy science in general and the person's interest in it in particular, by leaving academically valuable research that is not obviously marketable “in the corner” (Fig 2). The risk of industry's interests distorting research values also appeared in other stories about science over‐promising solutions and then not being able to deliver. A corollary was “complacency”, which stopped people from engaging with “urgent issues”, because of public engagements, such as television shows, in which science was “abused as a way of, almost, distracting [the public] so they do not have to worry about the destruction of the world, or environments, or anything; they do not have to do anything, because some clever scientist has got the problem solved”.

A number of participants addressed similar risks arising from the profit motive, worrying that this could destroy science in general and the person's interest in it in particular…

Finally, participants expressed concerns that their research might not live up to expectations or might fail the overall mission of the research centre. One participant made a model using only black bricks to symbolise that their research could perpetuate the use of fossil fuels rather than replace it. In contrast, direct material risk was usually seen as relating to the particular work carried out within the centre. While some participants modelled accidental release of bacteria or dangerous chemicals, the subsequent mitigation scenarios suggested that these were seen as taken care of by safety protocols and procedures. Of more concern appeared to be risks to the environment arising from the disposal of waste, such as plastic gloves and chemicals.

## Risk, fear and mitigation through communication

Overall, however, personal and systemic risks, such as fear of failure or being scooped, loomed much larger than material ones. Many participants used colourful metaphors and models, interpreting “risk” as fear or disillusionment, such as not getting expected results (“banging my head against the wall”), not completing their PhD (“trying to escape the Black Hole of Doom”), making a collaborator or somebody higher up angry when an experiment did not work (“sitting on the naughty step”) or messing up the experiment itself (“the wheels come off”). Participants also spoke about feeling lost (“left in the wilderness” or “digging oneself into the ground”) or severely depressed (“getting completely flattened” and “run over”). Discussion at one table turned to incidents where scientists had even taken their own lives: “It's something we don't talk about often enough, I think, but it's something that people cannot handle the stress of science. […] We don't really do much about it, it's just assumed, “Well, it's just a tough job”; but a lot of people cannot really handle it very well, and it's not accepted that you cannot do it well.”

Several respondents also connected these fears to the risk of becoming so involved in their work they might forget to make a life outside it: “I made it a little model of me being sad, fishing for results only getting a little shoe. Which is sad in itself, but I think the most important thing is that even if I fail in science I can do something else. The biggest fear and risk is to end up lonely, lonely on the planet.”

Overall, however, personal and systemic risks, such as fear of failure or being scooped, loomed much larger than material ones.

Talking was seen as one of the most important mitigation strategies, both to alleviate personal stress and pressure and to address institutional or systemic risks. Even those who had built models of material risks and modified them in ways to “contain” bacteria, or created technical solutions such as early warning sensors, a fire escape, or a machine that could take over dangerous tasks from humans, still highlighted the importance of “communicating about the practical risks of their work and making sure that everyone works responsibly”, often symbolised by two Lego people talking to each other. Collaboration and teamwork, communication, dissemination, public education and being open and honest about risks were all suggested as means of mitigation. But communication and collaboration within the laboratory were also modelled as a preferred strategy for mitigating risk stemming from the particular pressures of being an early‐stage researcher and as a means of reducing depression and isolation.

Thus, the material and technological risks (such as biosecurity) were seen by our participants as manageable by individual or group control and responsibility; in contrast, social, psychological, institutional and systemic risks (such as job security) were seen as much more unmanageable and beyond control. These insights are highly relevant in the context of debates about the industrialisation, marketisation and financialisation of the university sector, particularly in the UK. Responsibility for dealing with what was seen as an incentive system, in which research is increasingly about creating commercial products, appeared to be tacitly and indirectly allocated to what one might call research managers, or to research funders, policymakers and the government. However, this left the uncomfortable question of what responsibility scientists themselves should, or even could, exert over the field. As most of our participants were at the earlier stages of their careers, this may have been the most troubling finding of all, indicating—as the mental stress models appeared to show—a direct threat to the love that had brought them to science in the first place and undermining their confidence.

## Modelling responsibility in a new way

Conceived as a means of helping synthetic biologists better understand the risks of their work, the workshops instead revealed that mere technical threats that could lead to public rejection of a technology (at which RRI activities are mainly aimed) appear to have been adequately discussed and understood, at least within this particular research centre. However, RRI as deployed through the AREA (anticipate, reflect, engage and act) framework developed by the UK's Engineering and Physical Sciences Research Council's 7 does not yet adequately address the other risks identified: concerns about career progress in still‐emerging fields where experiments may fail more often than they succeed, and mental health risks from a lack of work–life balance. But there is also the unaddressed risk to the mission of science itself given the economic incentive system in which biotechnology research is situated and by framing biotechnology as responsible for saving the world 8.

We also urge paying more attention to what science policy can reasonably demand of the research workforce without crushing the curiosity and vitality of its postgraduate and postdoctoral participants

Importantly, this also raises questions about the RRI approach that is being used in these synthetic biology projects. The AREA framework is promoted in the UK by the EPSRC and BBSRC, who provide funding for the Centre, but the findings from our workshops suggest that this framework is not sufficient. In particular, it places the onus of responsibility on scientists without including reflection on how research managers, funders and science policymakers can better support them.

In this sense, it may be useful to draw upon the wider vision of the European Horizon 2020 version of RRI, which, although it is no less oriented towards marketable products and public acceptance, incorporates specific thematic elements beyond public engagement. In particular, the issue of “responsibility” in research and innovation includes taking care of the researchers and innovators within that system, particularly by those with the power to determine what constitutes publishable knowledge. We also urge paying more attention to what science policy can reasonably demand of the research workforce without crushing the curiosity and vitality of its postgraduate and postdoctoral participants, who are still, for all intents and purposes, in the apprenticeship phases of their education. However, while the most recent version of Horizon 2020 RRI 9 makes reference to institutions and institutional managers, it still does not address how individuals and institutions are supposed to balance demands from RRI protagonists concerned with science/society interactions against demands from political institutions, governments and industry for more products, patents and economic growth 10.

Although our sample size is too small to generalise, it is a concern that nearly all the participants who modelled an inability to talk about stress were male, suggesting that while women may be subject to more systemic exclusions, men may find it more difficult to build informal and interpersonal support systems.

**…**the particular risks faced by scientists in the early stages of the careers on large, potentially lucrative projects [..] need to be better understood and managed so as to prevent sacrificing their talent

Our workshops also made it clear that the particular risks faced by scientists in the early stages of the careers on large, potentially lucrative projects—which may fail through no fault of their own, but simply because failure is part of cutting‐edge science—need to be better understood and managed so as to prevent sacrificing their talent. As biotechnology as a field matures, extending “responsibility” to include discussing and mitigating risk and negative impact upon those tasked with carrying out its research should become an integral part of any RRI framework, considering the scientific workforce as an essential part of the public good. Policymakers, research funders and university administrators have both a responsibility and the opportunity to shape the innovation system in such a way that collaborative research for the public good can flourish and that the individuals tasked with developing technologies to address the grand challenges of our time are not bearing an undue burden, particularly at the beginning of their careers.

## Conflict of interest

The authors declare that they have no conflict of interest.
